# Inhibiting and Promoting Factors for the Use of Video Consultations Among Individuals Covered by Statutory Health Insurance in German Outpatient Care: Cross-Sectional Study

**DOI:** 10.2196/66027

**Published:** 2025-06-11

**Authors:** Lara Kleinschmidt, Jürgen Wasem, Udo Schneider, Anja Wadeck, Stephanie Sehlen, Sebastian Liersch, Katharina Schwarze, Franziska-Leonore Pankoke, Theresa Hüer

**Affiliations:** 1 Institute for Health Care Management and Research University of Duisburg-Essen Essen Germany; 2 Techniker Krankenkasse Hamburg Germany; 3 AOK Nordost Potsdam Germany; 4 AOK NordWest Dortmund Germany

**Keywords:** video consultation, survey, German outpatient medical care, inhibiting factors, promoting factors, perspective of insured individuals, telemedicine, VC, German, outpatient, medical care, health insurance, descriptive method, subgroup analysis, health technology, digital health

## Abstract

**Background:**

Video consultations (VC) have proven to be a useful tool to enhance access to medical care for patients. During the COVID-19 pandemic, the use of VC has risen sharply. However, since (the end of) the pandemic, they have only been used to a limited extent by insured individuals in the German statutory health insurance (SHI).

**Objective:**

The aim of this survey is to identify inhibiting and promoting factors for the use of VC in the SHI-insured population.

**Methods:**

Survey documents were distributed by 3 participating SHI funds to 33,816 insured individuals in 4 selected German federal states. Participation was anonymous and available in paper format or online via a QR code. Both descriptive methods as well as inferential statistics were performed for analysis. Subgroup analysis included evaluations based on gender, age groups, community size, chronic disease, and previous VC experience.

**Results:**

The response rate was 13.9%, resulting in 4600 included questionnaires from the 33,816 individuals approached. Although 75.3% (3132/4162) of the insured were interested in using VC in general, only 6.7% (302/4511) of them had used it at the time of the survey. Among respondents with little or no VC experience, 88.3% (2763/3129) stated that the lack of VC offered by physicians was the biggest obstacle to VC use. Other relevant inhibiting factors were concerns about the quality of medical care (1573/3589, 43.8%) and data protection (948/3861, 24.6%). A lack of technical equipment and a stable internet connection tended not to be an obstacle in the survey. Comparing subgroups, associations were identified in particular between the inhibiting factors and age groups as well as between the inhibiting factors and the presence of a chronic illness. With increasing age, participants were more likely to have data protection concerns (*P*<.001, Kendall Tau-c=0.128) or perceive VC as exhausting (*P*<.001, Kendall Tau-c=0.136). Similarly, participants with a chronic condition were more likely to perceive VC as stressful (chronic condition: 247/1177, 21% vs no chronic condition: 257/1847, 13.9%; χ²_2_=30.209, *P*<.001; Cramer V=0.1). The most relevant promoting factors were that a video application works without interruption (3624/3911, 92.7%) and that it is easy and intuitive to use (3674/3978, 92.4%).

**Conclusions:**

The results suggest that insured individuals are interested in using VC but were rarely offered VC appointments. Therefore, it is important to reduce potential obstacles on the part of the service providers, who are currently limiting the availability of VC. Existing hurdles can best be addressed by targeting subgroup-specific hurdles as they tend to vary between subgroups.

## Introduction

Telemedicine as an alternative health care delivery model uses digital technologies to overcome geographical barriers to health care provision, with the potential to enhance medical appointments and expand service accessibility [1]. In response to the COVID-19 pandemic, telemedicine services were widely used as a substitute for in-person consultations with medical professionals [2]. The most common applications of telemedicine in outpatient care were video consultations (VC) and telephone calls [3].

Before the COVID-19 pandemic, VC were hardly used in Germany [4]. However, with the decrease in reimbursement restrictions as well as the removal of usage field limitations that accompanied the onset of the COVID-19 pandemic, within one quarter, usage increased 6-fold to over 1 million VC in the second quarter of 2020 compared with the first quarter of 2020 [5]. Nevertheless, usage fell again toward the end of the pandemic. A report by the Statutory Health Insurance Fund (SHIF) BARMER showed monthly claims for the second quarter of 2022 that were more than 50% lower than during the pandemic waves in the second quarter of 2020 or the first quarter of 2021. They seemed to have stabilized at a relatively low level [6].

VC are most popular in the medical specialty of psychotherapeutic care. Other, less frequently used medical specialties are pediatrics, general practice, dermatology, and orthopedics. Indications were mainly for respiratory, musculoskeletal, and connective tissue diseases, as well as endocrine, nutritional, and metabolic disorders [5,6]. So far, VC have been used particularly by younger adults, women, employed persons [6,7], and people in urban regions [4,6,7].

In previous studies, patients often considered face-to-face visits to be the gold standard [8]. Further, a lack of experience with handling technical equipment and availability of technology seem to be hindering factors [8-10]. Privacy and data security have also been common concerns [10,11]. A current literature review on remote consultations in primary care during the pandemic highlighted the beneficial experience for patients in terms of convenience and reduced risk of infection, while concerns on privacy and data protection as well as technological skills or access to technology remained [12]. Research on patients’ perceptions on the use of telemedicine during COVID-19 in Germany confirms that, while some people rejected them, most patients are generally interested in using remote consultations [13]. Studies have further demonstrated the benefits of VC in terms of cost reduction and work-life balance, in particular for patients [14,15]. However, there seem to be differences in the user groups, as some participants responded to telemedicine with interest while others rejected it.

There is limited understanding regarding the rationale behind the low use of VC and the stagnation of VC use despite further digitalization after the COVID-19 pandemic. Thus, the aim of this study was to analyze which hurdles need to be overcome and to identify promoting factors to increase the use of VC. Particularly, the survey focused on the conditions – inhibiting and promoting factors – under which the insured accept the virtualization of their medical consultations. The study also examined VC user groups and medical fields of use.

## Methods

### Study Design

Based on preliminary work in the study, a standardized questionnaire was developed. Particularly, through a systematic literature review on the application settings for VC and the obstacles to its implementation, respective relevant aspects for the questionnaire were outlined and additionally discussed with 2 focus groups of insured individuals and evaluated with qualitative content analysis. A study protocol for the entire study has been published previously [16].

The questionnaire contains 3 sections: (1) previous use as well as possible barriers and facilitators to the use of VC, (2) preference survey using discrete choice experiments, and (3) sociodemographic and health-related information.

The questions to determine the outcomes were asked in the first section of the survey regarding knowledge of VC, previous VC use, frequency of VC use, inhibiting factors that may discourage VC use, and factors that promote VC use. Additionally, the first section inquired about the medical specialty in which VC was used or in which the respondent would prefer to receive VC, as well as suitable treatment occasions. Questions about potential barriers to VC could only be answered if no or 1 previous use was reported. As the results from the preference elicitation of the second section of the survey will be published separately, no further details are provided here. For subgroup analysis, exposure variables (ie, sociodemographic and health-related questions) were asked in the third section of the survey. These include overall health status, presence and type of chronic disease, and contact with physicians in the past 12 months.

The survey used categorical variables for most questions, except for frequency of use and age groups, which used an ordinal scale. Predefined factors about possible barriers and facilitators were asked on a 4-point Likert scale, and statements about appropriate treatment occasions were posed on a 5-point Likert scale. The possible barrier “I have not yet been offered a VC” was graphically embedded in the 4-point Likert scale but only with 2 possible answer options. Further, every Likert scale had an “I don't know” option, which was coded as missing during analysis. Those and 3 further questions also included open-text response options to enable wide exploration of potential responses. Overall, not all questions were mandatory, so missing responses were possible.

The design of the questionnaire underwent a pretest. A combination of think-aloud and probing methods [17] was used with 20 participants from various age groups and socioeconomic backgrounds. The survey took around 15 minutes to 20 minutes to complete. An excerpt of the survey can be found in Multimedia Appendix 1.

### Recruitment

The survey was sent to 33,816 randomly selected individuals living in 4 German regions (Westphalia Lippe, Mecklenburg-West Pomerania, Schleswig-Holstein, and Berlin) insured by the 3 participating SHIFs (Techniker Krankenkasse, AOK Nordost, AOK NordWest). Inclusion criteria for participation in the survey were being insured with one of the 3 participating SHIFs, age ≥18 years, and living in one of the 4 selected regions. Exclusion criteria were having a guardian, requiring high levels of care, linving in a nursing home, receiving palliative care, and a diagnosis of dementia. The exclusion of these individuals is based on the presumption that they would be incapable of answering independently or only to a limited extent. Further, people who refused the use of their data for research purposes were excluded [16].

The survey was conducted between November 2022 and March 2023. A reminder was sent after 2 months to increase the response rate.

### Ethical Considerations

This survey is part of the of a larger German research study entitled “Preference-based use of video consultations in urban and rural regions.” The financial resources for this investigation were provided by the Innovation Fund of the German Federal Joint Committee (funding number 01VSF20011). The study was subject to ethical approval by the Ethics Committee of the Medical Faculty at the University of Duisburg-Essen on September 27, 2022 (reference: 21-10283-BO). Participation in the survey was anonymous and could be conducted on paper or digitally via a QR code. The participants were required to sign a declaration of consent to complete the surveys. Further, they were informed about the purpose of the study and the processing of their data. To enhance the response rate, the option of participating in a raffle was made available, with the prize being vouchers valued at €50 (US $55.07) each.

### Statistical Analysis

The data were analyzed by descriptive as well as inferential statistics for quantitative evaluation. Descriptive data are presented as frequencies (n) and percentages (%). Likert scales were recoded from 4 levels (strongly agree; agree; somewhat disagree; disagree) to 2 levels (agree; disagree). Additionally, mean, median, and SD are reported for Likert scales. Participants who indicated “diverse” on the sex item were assigned to the female category in the context of this study. This was due to the small number of participants with this response. The allocation to the female category was made in adherence to the calculation standards of the SHIF [18], ensuring comparability.

Subgroup analyses included the exposure variables gender (male or female; age groups (18-29 years, 30-59 years, or ≥60 years); community size in reference to the classification “Stadt- und Gemeindetyp” as rural or urban by the Federal Institute for Research on Building, Urban Affairs and Spatial Development (rural community, small town, middle town, or large city) [19]; previous VC experience (yes or no), occupation (full-time employed, part-time employed, or not employed); and chronic disease (yes or no). The Pearson chi-square test was used to test whether there was an association between categorical variables, and the Cramer V value indicated the effect size [20]. Measures of correlation and the effect size for ordinal variables were calculated using Stuart-Kendall Tau-c [21]. The level of statistical significance was set at α=.05 (*P*≤.05). According to Cohen d, effect sizes that assume a value smaller than |0.1| are not relevant and were therefore not reported. Effect sizes greater than |0.1| to |0.3| are considered weak, sizes greater than |0.3| are considered moderate, and sizes greater than |0.5| are considered strong [22]. Statistical analyses were performed using the statistical software SPSS Version 28 (IBM Corp) [23].

## Results

### Response Rate and Participants’ Characteristics

Of the 33,816 insured persons the study questionnaire was sent to by the participating SHIFs, 32,993 could be reached, and 823 questionnaires were returned for various reasons. A reminder was sent to 24,589 insured after 4 weeks. Between December 2022 and March 2023, 4715 people returned the questionnaires: 61.9% (2919/4715) responded online, and 38.1% (1796/4715) replied to the paper version, resulting in a response rate of 13.9% (4715/33,816). After excluding 115 completely blank questionnaires, 4600 respondents were included in the analysis.

Table 1 summarizes the sociodemographic characteristics of the respondents as well as data on the basic population. The final sample consisted of slightly more female participants (2370/4239, 55.9%), with approximately one-half of the participants belonging to the age group of 30 years to 59 years (2205/4240): 34.7% (1473/4240) of the participants were older than 60 years, while 13.3% (562/4240) were aged between 18 years and 29 years. Most of the participants were from urban areas (large city: 1787/4186, 42.7%). Full-time employment (1842/4180, 44.1%) and not being employed (1491/4180, 35.7%) were the most common responses regarding occupation. For a comparison with the basic population, data for the fourth quarter of 2023 were requested from the SHIFs on their insured who met the inclusion and exclusion criteria. Compared with this basic population, the sample included a slightly higher proportion of women (2370/4239, 55.9% vs 2,500,814/4,940,741, 50.6%). The age distribution was similar for those aged 30 years to 59 years (2205/4240, 52% vs 2,629,478/4,940,741, 53.2%), but the study included more older participants (≥60 years: 1473/4240, 34.7% vs 1,320,362/4,940,741, 26.7%) and slightly fewer younger participants (18-29 years: 562/4240, 13.3% vs 911,770/4,940,741, 18.5%).

**Table 1:** Sociodemographic characteristics of participants and comparison with basic population, with data provided by the participating Statutory Health Insurance Funds (SHIFs).

Characteristics	Participants (n=4600), n (%)	Comparison population (n=4,940,741), n (%)^a^	
**Sex (n=4239)**			
	Female, including diverse	2370 (55.9)	2,500,814 (50.6)
	Male	1869 (44.1)	2,439,927 (49.4)
**Age group (years; n=4240)**			
	18-29	562 (13.3)	911,770 (18.5)
	30-59	2205 (52)	2,629,478 (53.2)
	≥60	1473 (34.7)	1,320,362 (26.7)
**Community size (n=4186)**			
	Rural community	778 (18.6)	—^b^
	Small town	761 (18.2)	—
	Middle town	860 (20.5)	—
	Large city	1787 (42.7)	—
**Occupation (n=4180)**			
	Full-time employed	1842 (44.1)	—
	Part-time employed	847 (20.3)	—
	Not employed	1491 (35.7)	—

^a^Population covered by participating SHIFs in the fourth quarter 2023 who met the inclusion and exclusion criteria.

^b^Not available.

Regarding self-rated health information (see Table 2), a large proportion of the participants reported a “good” (2029/4194, 48.4%) or “very good” (851/4194, 20.3%) self-perceived health status. However, 40.9% (1689/4128) of participants report having a chronic disease. The majority of participants had visited their general practitioner (GP; 3729/4101, 90.9%) or another medical specialist (3258/3944, 82.6%) at least once in the past 12 months. However, few (630/3694, 17.1%) had seen a psychiatrist or psychotherapist professional within the past 12 months.

**Table 2:** Participants’ self-rated health data.

Health characteristics		Participants, n (%)
**Overall health status (n=4194)**		
	Very good	851 (20.3)
	Good	2029 (48.4)
	Medium	1098 (26.2)
	Bad	190 (4.4)
	Very bad	26 (0.6)
**Chronic disease (n=4128)**		
	Yes	1689 (40.9)
	No	2439 (59.1)
**Contact with a general practitioner in the past 12 months (n=4101)**		
	No contact in the past 12 months	372 (9.1)
	At least 1 contact in the past 12 months	3729 (90.9)
**Contact with physicians of other specialties in the past 12 months (n=3944)**		
	No contact in the past 12 months	686 (17.4)
	At least 1 contact in the past 12 months	3258 (82.6)
**Contact with** **psychiatrists or** **psychotherapists in the past 12 months (n=3694)**		
	No contact in the past 12 months	3064 (82.9)
	At least 1 contact in the past 12 months	630 (17.1)

### Interest in Use of VC

Although 74.7% (3375/4520) of the participants were aware of VC, only 6.7% (302/4511) of the sample were VC users. Of those, 154 used VC only once, 51 used it twice, and 95 used it more than twice. Significant associations with age (χ²_2_=51.67, *P*<.001; Cramer V=0.111), community size (χ²_3_=55.220, *P*<.001; Cramer V=0.115) and contact with a psychiatrist or psychotherapist (χ²_1_=83.455, *P*<.001; Cramer V=0.151) were identified with VC use. Participants in the younger or middle age groups were more likely to have previous experience with VC use compared with those older than 60 years (18-29 years: 55/560, 9.8%; 30-59 years: 177/2181, 8.1%; ≥60 years: 41/1451, 2.8%). Additionally, participants living in large cities were more likely to have experience with VC use compared with those in rural communities (rural community: 25/770, 3.2%; small town: 35/770, 4.7%; middle town: 37/851, 4.3%; large city: 173/1769, 9.8%). In addition, people who had received psychotherapy at least once in the past 12 months were more likely to have experience with VC than people who had not received psychotherapeutic care in the last 12 months (at least 1 contact: 97/615, 15.8% vs no contact: 164/3047, 5.4%).

When the insured who had already used VC at least twice (n=146) were asked about the physicians with whom they most commonly used VC, 143 responded and provided 151 responses (more than one response was possible). The most commonly selected physicians were psychotherapists, psychiatrists, or neurologists (72/143, 50.3%) as well as GPs (63/143, 44.1%). VC experience with physicians from other specialties was rather rare (orthopedists: 8/143, 5.6%; dermatologists: 5/143, 3.5%; gynecologists: 3/143, 2.1%).

Of the insured, 75.3% (3132/4162) expressed interest in potentially using VC. A clear age gradient was evident, with younger age groups being more likely to show interest in VC use (18-29 years: 485/548, 88.5%; 30-59 years: 1789/2166, 82.6%; ≥60 years: 852/1429, 59.6%). A chi-square test showed a significant association (χ²_2_=303.396, *P*<.001; Cramer V=0.271). When asked which physicians they would like to use VC with, 4135 responded and provided 12,821 responses (more than one response was possible). The most frequently selected physicians were GPs (3693/4135, 89.3%), psychotherapists and psychiatrists (1572/4135, 38%), surgeons (for pre- and postsurgery consultations; 1432/4135, 34.6%), and dermatologists (1339/4135, 32.4%). There were significant dependencies in the demand for services from GPs between age groups (χ²_2_=94.388, *P*<.001; Cramer V=0.149) and employment levels (χ²_2_=133.773, *P*<.001; Cramer V=0.179). The survey results indicated that individuals in the age groups of 18 years to 29 years (501/562, 89.1%) and 30 years to 59 years (1911/2205, 86.7%) were more inclined to participate in virtual consultations with their GP than the older age group (≥60 years: 1113/1473, 75.6%). Moreover, participants with full-time (1638/1842, 88.9%) or part-time (742/847, 87.6%) employment were more likely to consider participating in VC with their GP than participants without employment (1114/1491, 74.7%).

Regarding the demand of VC services in psychotherapeutic care, the differences among people with a previous experience with using VC should be emphasized. They were significantly more likely to want psychotherapy services to be offered via VC (175/302, 57.9%) than people who had never used VC (1391/4209, 33%). The chi square test was significant (χ²_1_=77.079, *P*<.001; Cramer V=0.131). Further, a significant association was observed for different age groups (χ²_2_=273.412, *P*<.001; Cramer V=0.254), as participants in the age groups of 18 years to 29 years (278/562, 49.5%) and 30 years to 59 years (950/2205, 43.1%) were more likely to seek psychotherapeutic care via VC than participants older than 60 years (283/1473, 19.2%). Regarding differences in occupation, full-time (756/1842, 41%) and part-time workers (377/847, 44.5%) were more likely than the unemployed (364/1491, 24.4%) to seek psychotherapy through VC, which was also significantly correlated (χ²_2_=134.075, *P*<.001; Cramer V=0.179).

### Inhibiting Factors

Results on possible hindering factors only included participants with no or only 1 previous VC experience: 88.3% (2763/3129) reported that a barrier to not using VC was not being offered VC by their physician. Other relevant hindering factors were concerns about lower quality of care (1573/3589, 43.8%) and data protection (948/3861, 24.6%). In addition, 33.9% (166/489) of non-native speakers reported language barriers as a hindering factor for VC use. In contrast, technical issues such as inadequate equipment (544/4052, 13.4%) or lack of knowledge about how to use it (698/4064, 17.2%) as well as poor internet connectivity (538/3908, 13.8%) did not appear to be major obstacles for the insured population.

It is also relevant to note that, when looking at the median for all potential barriers (except “I have not yet been offered a video consultation”), the statements were not considered to be major barriers for respondents overall, as the median for barriers ranged from 1 “disagree” to 2 “somewhat disagree” (see Table 3). Therefore, subgroup analyses were carried out to provide a differentiated view of potential barriers within individual user groups.

**Table 3:** Inhibiting factors for video consultation use (mean, median, and SD).

Factors	Responses, n	Mean (SD)	Median	Minimum-maximum
I have not yet been offered a video consultation.	3129	N/A^a^	N/A	1-2
My internet connection isn't good.	3908	1.54 (0.865)	1	1-4
I don't have the technical equipment.	4052	1.45 (0.9)	1	1-4
I lack experience in handling technical devices.	4064	1.57 (0.923)	1	1-4
I fear that the quality of medical care will suffer.	3589	2.33 (0.993)	2	1-4
I have concerns about data protection.	3861	1.93 (0.971)	2	1-4
I struggle to communicate in German language.^b^	489	2.054 (1.173)	2	1-4
I find video consultations too exhausting.	3279	1.68 (0.906)	1	1-4

^a^Not applicable.

^b^Analyzed only for people who stated a language other than German as their native language and did not state German as their native language (n=489).

Significant correlations in the assessment of the inhibiting factors were identified by age group (“I have concerns about data protection”: *P*<.001, Kendall Tau-c=0.128; “I find video consultations too exhausting”: *P*<.001, Kendall Tau-c=0.136). With increasing age, participants were more likely to state that they perceived data protection concerns as a hurdle (18-29 years: 93/502, 18.5%, 30-59 years: 400/1926, 20.8%; ≥60 years: 392/1214, 32.3%). In addition, participants older than 60 years were more likely to perceive VC as stressful (18-29 years: 58/448, 13%; 30-59 years: 206/1677, 12.3%; ≥60 years: 254/962, 26.4%). In particular, technical issues such as a poor internet connection (*P*<.001, Kendall Tau-c=0.122), technical equipment (*P*<.001, Kendall Tau-c=0.206), and experience with handling technical equipment (*P*<.001, Kendall Tau-c=0.282) showed significant correlations by age group as they were perceived as greater obstacles the older the participant was (see Multimedia Appendix 2). Similarly, for respondents with chronic conditions, significant relationships were found for technical issues (“My Internet connection isn't good”: χ²_3_=47.161, *P*<.001; Cramer V=0.114, *P*<.001; “I don't have the technical equipment”: χ²_3_=117.426, *P*<.001; Cramer V=0.178; “I lack experience in using technical devices”: χ²_3_=151.086, *P*<.001; Cramer V=0.201). In addition, VC were significantly more likely to be perceived as stressful when participants had a chronic condition, with a significant association observed (chronic condition: 247/1177, 21% vs no chronic condition: 257/1847, 13.9%; χ²_3_=30.209, *P*<.001; Cramer V=0.1). Significant associations could also be seen when analyzing the subgroups by occupation (“My Internet connection isn’t good”: χ²_6_=184.695, *P*<.001; Cramer V=0.159; “I don't have the technical equipment: χ²_6_=369.659, *P*<.001; Cramer V=0.221; “I lack experience in handling technical devices”: χ²_6_=486.274, *P*<.001; Cramer V=0.253; “I find video consultations too exhausting”: χ²_6_=164.01, *P*<.001; Cramer V=0.164). In terms of technical barriers, unemployed participants were more likely to report a poor internet connection (unemployed: 288/1240, 23.2% vs part-time employed: 72/749, 9.6% or full-time employed: 142/1662, 8.5%), a lack of technical equipment (unemployed: 348/1333, 26.1% vs part-time employed: 42/763, 5.5% or full-time employed: 109/1681, 6.5%), or a lack of experience with using technical equipment (unemployed: 441/1337, 33% vs part-time-employed: 71/764, 9.3% or full-time employed: 125/1686, 7.4%). Further, compared with part-time employed participants (82/630, 13%), those who were not employed (276/997, 27.7%) were more likely to perceive VC as stressful. Among non-native speakers, those who were not employed were more likely to state that they had difficulty speaking German with their physician via video (unemployed: 70/161, 43.5% vs part-time employed: 26/97, 26.8% or full-time employed: 67/227, 29.5%; χ²_6_=27.280, *P*<.001; Cramer V=0.168). The analysis of the barriers according to the community size showed a significant correlation regarding internet connection. Respondents in smaller communities stated having internet problems more often than respondents living in larger cities (rural community: 154/689, 22.4%; small town: 117/657, 17.8%; middle town: 78/744, 10.5%; large city: 147/1556, 9.4%; *P*<.001; Kendall Tau-c=–0.100). There were no significant associations in the gender subgroup. See Multimedia Appendix 2 for details on correlation and association measures and effect sizes.

### Promoting Factors

Nearly all respondents agreed that a video application that “works without interruptions” (3624/3911, 92.7%) and is “easy and intuitive to use” (3674/3987, 92.4%) is important for VC. The survey results also indicated that a video application that can be used by people with visual, hearing, or mobility impairments (2998/3574, 83.9%), information about data protection (3194/3822, 83.6%), and information provided by their physician (3258/3915, 83.2%) are important for VC use. Additionally, 78.1% (3043/3898) of the sample considered a video application with many features to be relevant. For 81.0% (404/499) of non-native speakers support in the conversation in the respective language was a relevant driver. Finally, 70.9% (2625/3701) of the participants considered information services, such as a telephone hotline for technical questions or brochures, to be important for VC use. The proportion of people who answered “strongly agree” or “agree” was thus greater than 70% for all statements.

The strong agreement with all aspects was also evident when looking at the medians, which ranged between 3 and 4 for all statements (see Table 4). Since the majority of respondents rated all aspects of the survey as important, it was useful to analyze possible differences between subgroups.

**Table 4:** Promoting factors for video consultation use (mean, median, and SD).

Factors	Responses, n	Mean (SD)	Median	Minimum-maximum
Information services	3701	2.8 (0.915)	3	1-4
Information from my physician about aspects to consider during the video consultation	3915	3.07 (0.825)	3	1-4
Easy/intuitive operation of the video application	3978	3.44 (0.764)	4	1-4
Video application with many features	3898	3.06 (0.892)	3	1-4
Video application works without interruptions	3911	3.47 (0.752)	4	1-4
Can also be used by people with visual, hearing or movement impairments	3574	3.22 (0.888)	3	1-4
Information about data protection	3822	3.12 (0.841)	3	1-4
Conversation support in the native language^a^	499	3.17 (0.923)	3	1-4

^a^Analyzed only for people who stated a language other than German as their native language and did not state German as their native language (n=499).

The subgroup analysis revealed significant correlations for some potentially promoting factors by age group (Easy/intuitive operation of the video application: *P*<.001, Kendall Tau-c=–0.104; Video application with many features: *P*<.001, Kendall Tau-c=–0.131; Video application works without interruptions: *P*<.001, Kendall Tau-c=–0.143; Can be used by people with visual, hearing, or mobility impairments: *P*<.001, Kendall Tau-c=–0.147; Conversation support in native language: *P*<.001, Kendall Tau-c=–0.170). Older participants were less likely to consider it important for the video application to be easy/intuitive to use (18-29 years: 495/525, 94.3%; 30-59 years: 1948/2065, 94.3%; ≥60 years: 1100/1240, 88.7%), that the VC has many features (18-29 years: 456/533, 85.6%; 30-59 years: 1645/2024, 81.3%; ≥60 years: 839/1197, 70.1%), or works without interruptions (18-29 years: 514/536, 95.9%; 30-59 years: 1938/2040, 95%; ≥60 years: 1040/1187, 87.6%). Older people were also less likely to consider the ease of use of the video application for people with visual, hearing, or mobility impairments as important (18-29 years: 456/504, 90.5%; 30-59 years: 1600/1848, 86.6%; ≥60 years: 826/1082, 76.3%). Further, among non-native speakers, younger age groups (87/98, 89%) were more likely to agree that support in their respective native language is important than middle-aged participants (261/320, 81.6%) or participants older than 60 years (53/78, 68%). The subgroup analysis regarding occupation indicated similar results, as significant relationships were identified for the importance of a video application with many features (χ²_6_=106.685, *P*<.001; Cramer V=0.12), easy usability (χ²_6_=122.065, *P*<.001, Cramer V=0.127), usability for people with impairments (χ²_6_=100.166, *P*<.001, Cramer V=0.121), and an application without interruptions (χ²_6_=181.007, *P*<.001, Cramer V=0.156). Fully employed (1408/1712, 82.2%) as well as part-time employed (626/782, 80.1%) persons tended to perceive having many features as more relevant than unemployed people (877/1223, 71.7%). Participants who worked full-time or part-time were more likely to rate an easy and intuitive video application to use as important (full-time employment: 1645/1736, 94.8% or part-time employment: 760/797, 95.4% vs unemployed: 1104/1260, 87.6%). Similarly full- and part-time workers were more likely to rate the application’s ability to be used by people with visual, hearing, or mobility impairments (full-time employment: 1362/1559, 87.4% or part-time employment: 643/729, 88.2% vs unemployed: 851/1110, 76.7%) and its ability to work without interruptions (full-time employment: 1640/1724, 95.1% or part-time employment: 774/792, 97.7% vs unemployed: 1043/1209, 86.3%) as supporting factors, compared with participants who are unemployed. The findings further showed associations between the importance of the application to have many features and having a chronic condition (χ²_3_=39.493, *P*<.001; Cramer V=0.104). Respondents with a chronic condition stated significantly more frequently that a video application with many features is important (chronic condition: 1102/1470, 75% vs no chronic condition: 1781/2211, 80.6%). There were no significantly relevant correlations or associations among the subgroups based on community size or previous VC experience. See Multimedia Appendix 3 for details on correlation and association measures and effect sizes.

## Discussion

### Findings on Inhibiting and Promoting Factors for VC Use

This study provides important insights into the usage behavior and possible existing barriers and facilitating factors for VC use after the COVID-19 pandemic. The self-reported use of VC among the insured population was rather low, at 6.7%. Younger individuals and people living in large cities were the main user groups. The most common medical specialty in which VC were used was psychotherapeutic and psychiatric care, even though most insured persons would like to use VC with their GP. Another preferred medical specialty for VC use was dermatological care and pre and postsurgery consultations. The user groups identified here aligned with those identified in other studies [4,6,7,24]. According to a claims data analysis in the same 4 regional areas, covering the time period from April 2017 to December 2020, young people and those residing in urban areas were more likely to use VC [7]. Additionally, psychotherapeutic care was shown to be the specialty VC are being used most frequently in both the claims data analysis and this study. However, there may be some bias in this result, as people from larger cities tend to use psychotherapy more often [6]. Nevertheless, we can conclude that there were no significant changes in the user groups during and after the pandemic.

This study further confirms that the younger generation and people living in urban environments are more motivated to use VC (previously and potentially in future times) instead of older age groups or people residing in rural areas. For these groups, however, VC may be particularly useful. VC have been identified as potentially beneficial for improving access to medical care in rural areas [25]. In addition, older people (often with a chronic condition) could benefit from more immediate contact with the physician for multiple routine checkups that require less effort (eg, travel and waiting time). The results also suggest that VC are beneficial for full or part-time employed people as they would like to be offered those services more often. Yet, older participants, participants with a chronic condition, and unemployed people in particular report difficulties with the technical requirements or handling thereof as well as perceiving VC as stressful.

Regarding barriers to VC use, the lack of services offered is by far the biggest obstacle, alongside all other aspects surveyed (eg, lack of technical equipment, poor internet connection, data protection, quality of care). This finding is in line with the results of previous studies stating the lack of services offered by GPs as a reason for the discrepancy between patients’ willingness to use services and their actual use [26,27]. For providers, studies suggest that there may be a greater administrative burden or that they are only willing to offer VC for specific types of treatment (eg, checkups) as part of their regular practice [28,29]. Although preliminary insights into the providers’ perspectives and potential explanations for their reservations exist, further investigation is necessary to provide a more comprehensive understanding of the issue.

Another important outcome of the study is that technical difficulties (ie, lack of technical equipment, insufficient knowledge of how to use technical equipment, poor internet connection) have become less relevant. Nonetheless, it is important to consider that, when they do occur, they can significantly disrupt the user experience [9,27]. Additionally, the study identified that people with previous experience with VC were also more likely to seek care via remote consultation. This suggests that concerns hindering VC use may decrease with increasing experience and familiarity. The findings of Mueller et al [11] indicate that the feasibility and appropriateness of VC are easier to assess for individuals with VC experience and that the benefits outweigh the concerns. Perceived obstacles to usage do not vary based on place of residence (rural or urban regions) but rather by age group and employment level.

Regarding promoting factors, the participants placed great importance on factors that contribute to the successful use of the video application (eg, no interruptions; easy use; usability for people with visual, hearing, or movement impairments). This trend was especially noticeable among subgroups based on age and occupation, as younger people and, in particular, part-time employed participants placed more value on these aspects. It was also notable that people with a chronic condition were significantly more likely to consider a video application with many features to be important. Studies show that VC are suitable for people with chronic conditions [30,31]. Considering the frequent routine examinations required, along with further digitization including reporting of health-related data or images, virtual conferences could benefit this population.

In the light of the results, the implementation of targeted strategies may be required to appeal to specific groups. Possible approaches could be targeted information services provided by medical practitioners and the professional public, as the majority of respondents were in favor of such services. One potential strategy to enhance the use of VC in clinical practice could be to educate the identified target groups on the benefits. Furthermore, the provision of information on the technical aspects of VC in more accessible languages and formats (eg, age-adequate) as well as on the application possibilities for chronically ill individuals could support more sustainable, interruption-free use and eventually reduce perceived barriers.

### Limitations

It is important to consider the possible limitations of the study when interpreting the results. A response rate of 13.9% is considered satisfactory, in particular in light of the relatively large sample size (n=4600). This study does not include a nationwide sample but individuals from only certain regions. The insured persons of the 3 SHIFs in the included regions represented 6.7% of the total Statutory Health Insurance (SHI) population. To mitigate the potential bias that SHIFs could introduce distortions due to their patient characteristics, different types of SHIFs were included, as well as SHIFs in both urban and rural regions. Regarding participant characteristics, the random selection of study participants should reflect the basic population in terms of age, sex, and place of residence. There were no significant differences between the sample and the basic population regarding sex. However, individuals with an interest in VC or health care (eg, due to age-related differences) might be more likely to respond to the questionnaire. The survey received a higher response rate from older individuals (age group ≥60 years), which may be explained by their greater need for health care services and related increased interest in health surveys. Moreover, the possibility to participate online via a QR code and on paper was included to counteract the exclusion of off-liners, who tend to be in the older age groups. Since the proportion of participants in this age group was slightly higher than in the basic population, as aforementioned, the exclusion of nontechnical individuals should be insignificant. Nevertheless, individuals who do not have a strong interest in VC may be underrepresented. Therefore, subgroup analyses were used to analyze the perspectives of the different groups. It should be noted that the subgroups of age and employment cannot be clearly distinguished from each other and show overlapping cases, as most people older than 60 years are also retired and therefore counted as not employed.

Additionally, it is impossible to completely rule out systematic errors. In this study, a tendency toward the middle or nondifferentiation in questions could occur in response behavior due to the use of item batteries (eg, Likert scales). Further, there may have been some issues with the item “lack of VC offered” (2-level nominal scale) graphically embedded in a Likert scale, which deviates from the traditional Likert scale. Upon examining the results of the Likert scales for hindering factors, it is evident that the medians were mostly in the range of 1 to 2. This may suggest that the hindering factors were either insignificant or not pronounced. However, such an assumption can be misleading because it may overlook certain subgroups in the data. Identifying these subgroups is crucial for developing targeted solutions. With regard to the facilitating factors, it can be seen that most factors were considered important. Further studies that weigh the factors against each other may be more helpful here. Concerning measures of correlation, in most cases, the calculated effect sizes were, according to Cohen, categorized as weak, as they ranged from |0.1| to |0.3|.

Finally, a limitation arose from the fact that the experience of privately insured persons was not considered. This approach mirrors the design of the German system, where approximately 90% of the population is covered by SHI. In addition, people requiring high levels of care, nursing home residents, people receiving palliative care, and people with cognitive impairments were not part of this study. Consequently, subsequent research may concentrate on these groups, as there are indications that telemedicine could prove particularly advantageous in these contexts [32,33].

### Conclusion

The user groups for VC use before and after the COVID-19 pandemic have remained unchanged. The analysis identified that hurdles differ depending on the subgroups and are particularly more present among those who could benefit most from them, including older people or individuals living with a chronic condition. Conversely, young people and those residing in urban areas exhibited a higher propensity to use VC. Understanding the reasons for the perceived hurdles and specifically addressing them as well as further subgroup target-specific interventions can lead to enhanced use. However, factors contributing to nonadoption are more likely to be on the provider side, as insured people are interested in receiving VC. Therefore, the perspectives of providers and their barriers should be explored in further research.

## Appendix

Multimedia Appendix 1 Excerpt of the survey.

Multimedia Appendix 2 Correlation/association and effect size of inhibiting factors.

Multimedia Appendix 3 Correlation/association and effect size of promoting factors.

## Abbreviations

GP: general practitioner
SHI: Statutory Health Insurance
SHIF: Statutory Health Insurance Fund
VC: video consultations

## Data Availability

Primary data of the survey are not publicly available due to data protection reasons.
