# Supplementary material for: Inhibiting and Promoting Factors for the Use of Video Consultations Among Individuals Covered by Statutory Health Insurance in German Outpatient Care: Cross-Sectional Study
Source: J Med Internet Res. 2025 Jun 11;27:e66027. doi: 10.2196/66027 (PMC12198700; doi:10.2196/66027)
Supplement: Multimedia Appendix 1 [file jmir_v27i1e66027_app1.docx]

## **Appendix 1:** Excerpt of the survey

**Erfahrungen und Einstellungen zur Videosprechstunde**

1. **Haben Sie die Videosprechstunde mit einem Arzt/einer Ärztin schon genutzt?**

|  | ja, bereits **drei oder mehr** Videosprechstunden | 🡪 weiter mit **Frage 4** |
| --- | --- | --- |
|  | ja, bereits **zwei** Videosprechstunden |  |
|  | ja, aber erst **eine** Videosprechstunde | 🡪 weiter mit **Frage 3** |
|  | nein, noch nie |  |

1. **Was hat Sie bisher daran gehindert, Videosprechstunden zu nutzen?**

**[Bitte setzen Sie in jeder Zeile genau ein Kreuz.]**

|  | **trifft gar nicht zu** | **trifft eher nicht zu** | **trifft zu** | **trifft voll zu** | **weiß nicht** |
| --- | --- | --- | --- | --- | --- |
| Mir wurde bisher **keine Videosprechstunde angeboten**. |  |  |  |  |  |
| Meine **Internetverbindung** ist nicht gut. |  |  |  |  |  |
| Mir fehlt die **technische Ausstattung** (z.B. Smartphone, Laptop mit Kamera). |  |  |  |  |  |
| Mir fehlt die **Erfahrung in der**  **Bedienung technischer Geräte.** |  |  |  |  |  |
| Ich befürchte, dass die **Qualität der**  **ärztlichen Versorgung** darunter leidet. |  |  |  |  |  |
| Ich habe **Bedenken beim Datenschutz**. |  |  |  |  |  |
| Es fällt mir schwer, in der **deutschen Sprache** **per Video** mit einem Arzt/einer Ärztin zu sprechen. |  |  |  |  |  |
| Ich finde Videosprechstunden **zu**  **anstrengend**. |  |  |  |  |  |
| sonstiger Grund:  [Bitte im Feld rechts eintragen.] |  | | | | |
| 🡪 weiter mit **Frage 5** | | | | | |
|  | | | | | |

1. **Mit welchem Arzt/welcher Ärztin hatten Sie schon eine Videosprechstunde?**

**[Mehrfachantworten sind möglich.]**

|  | Hausarzt/-ärztin | |
| --- | --- | --- |
|  | Psychotherapeut/-in, Psychiater/-in, Nervenarzt/-ärztin | |
|  | Frauenarzt/-ärztin | |
|  | Hautarzt/-ärztin | |
|  | Orthopäde/-in | |
|  | sonstiger Arzt/sonstige Ärztin:  [Bitte im Feld rechts eintragen.] |  |

1. **Bei welchem Arzt/welcher Ärztin würden Sie Leistungen auch per Videosprechstunde in Anspruch nehmen wollen?**

**[Mehrfachantworten sind möglich.]**

|  | Hausarzt/-ärztin | |
| --- | --- | --- |
|  | Psychotherapeut/-in, Psychiater/-in, Nervenarzt/-ärztin | |
|  | Frauenarzt/-ärztin | |
|  | Urologe/-in | |
|  | Hautarzt/-ärztin | |
|  | Orthopäde/-in | |
|  | Hals-Nasen-Ohren-Arzt/-Ärztin | |
|  | Chirurg/-in  (z.B. für Gespräche vor/nach Operationen, Kontrolltermine) | |
|  | Augenarzt/-ärztin | |
|  | Lungenfacharzt/-ärztin | |
|  | Onkologe/-in (Krebserkrankungen) | |
|  | Sonstiger Arzt/sonstige Ärztin:  [Bitte im Feld rechts eintragen.] |  |

1. **Was wäre Ihnen wichtig, bei der Nutzung von Videosprechstunden?**

**[Bitte setzen Sie in jeder Zeile genau ein Kreuz.]**

|  | **stimme nicht zu** | **stimme eher nicht zu** | **stimme zu** | **stimme voll zu** | **weiß nicht** |
| --- | --- | --- | --- | --- | --- |
| **Informationsangebote** (z.B. Telefonhotline bei Technikfragen, Broschüre) |  |  |  |  |  |
| **Informationen durch meinen Arzt/ meine Ärztin**, worauf ich in der Videosprechstunde achten muss |  |  |  |  |  |
| **einfache/intuitive** **Bedienung** des  Videoprogramms (bzw. Software) |  |  |  |  |  |
| **Videoprogramm** **mit vielen Funktionen** (z.B. Chat, Anzeigen von Laborergebnissen) |  |  |  |  |  |
| **Videoprogramm** funktioniert ohne Unterbrechungen („kein Ruckeln“) |  |  |  |  |  |

|  | **stimme nicht zu** | **stimme eher nicht zu** | **stimme zu** | **stimme voll zu** | **weiß nicht** |
| --- | --- | --- | --- | --- | --- |
| **Bedienung** des Videoprogramms ist auch **für Personen mit Seh-, Hör- oder Bewegungseinschränkung** gut möglich |  |  |  |  |  |
| Aufklärung über den **Datenschutz** |  |  |  |  |  |
| Falls Deutsch nicht die Muttersprache ist:  **Unterstützung** im Gespräch mit meinem Arzt/meiner Ärztin **in meiner Muttersprache** (z.B. Untertitel) |  |  |  |  |  |
| Sonstiges:  [Bitte im Feld rechts eintragen.] |  | | | | |

**Angaben zu Ihrer Person und zu Ihrem Gesundheitszustand**

1. **Welches Geschlecht haben Sie?**

|  | männlich |
| --- | --- |
|  | weiblich |
|  | divers |

1. **Welcher Altersgruppe gehören Sie an?**

|  | 18 bis 29 Jahre |
| --- | --- |
|  | 30 bis 39 Jahre |
|  | 40 bis 49 Jahre |
|  | 50 bis 59 Jahre |
|  | 60 bis 69 Jahre |
|  | 70 bis 79 Jahre |
|  | 80 Jahre oder älter |

1. **Was ist Ihre Muttersprache?** **[Mehrfachantworten sind möglich.]**

|  | Deutsch |  | Bosnisch/Kroatisch/Montenegrinisch/Serbisch | |
| --- | --- | --- | --- | --- |
|  | Russisch |  | Arabisch | |
|  | Türkisch |  | Bulgarisch | |
|  | Polnisch |  | Rumänisch | |
|  | Italienisch |  | Ungarisch | |
|  | Englisch |  | Griechisch | |
|  | Spanisch |  | andere:  [Bitte rechts eintragen.] |  |

1. **Sind Sie derzeit berufstätig?**

|  | ja, ganztags |  | nein, Hausfrau/Hausmann |
| --- | --- | --- | --- |
|  | ja, in Teilzeit |  | nein, Renten-/Pensionsbezug |
|  | ja, Mini/Midi-Job |  | nein, arbeitssuchend |
|  |  |  | nein, sonstiger Grund |

1. **In welchem Bundesland leben Sie?**

|  | Berlin |
| --- | --- |
|  | Mecklenburg-Vorpommern |
|  | Nordrhein-Westfalen |
|  | Schleswig-Holstein |

1. **Wie würden Sie den Ort, an dem Sie wohnen, beschreiben?**

[Hinweis: „In der Nähe“ meint, dass die Großstadt (mindestens 100.000 Einwohner) in ca. 30 Minuten mit dem Auto erreichbar ist.]

|  | Landgemeinde (weniger als 5.000 Einwohner **mit** Großstadt in der Nähe) |
| --- | --- |
|  | Landgemeinde (weniger als 5.000 Einwohner **ohne** Großstadt in der Nähe) |
|  | Kleinstadt (5.000 bis 20.000 Einwohner **mit** Großstadt in der Nähe) |
|  | Kleinstadt (5.000 bis 20.000 Einwohner **ohne** Großstadt in der Nähe) |
|  | Mittelstadt (über 20.000 bis 100.000 Einwohner **mit** Großstadt in der Nähe) |
|  | Mittelstadt (über 20.000 bis 100.000 Einwohner **ohne** Großstadt in der Nähe) |
|  | Großstadt (mehr als 100.000 Einwohner) |

1. **Wie ist Ihr Gesundheitszustand im Allgemeinen?**

|  | sehr gut |
| --- | --- |
|  | Gut |
|  | Mittelmäßig |
|  | Schlecht |
|  | sehr schlecht |

1. **Leiden Sie an einer chronischen Erkrankung?**

[Hinweis: Als chronische Erkrankungen werden lang andauernde Krankheiten bezeichnet, die nicht vollständig geheilt werden können, z.B. Diabetes, Rheuma, Asthma.]

|  | ja | 🡪 weiter mit **Frage 23** |
| --- | --- | --- |
|  | nein | 🡪 weiter mit **Frage** **24** |

1. **Unter welcher chronischen Erkrankung leiden Sie?**

**[Mehrfachantworten sind möglich.]**

|  | rheumatoide Arthritis/Rheuma | |
| --- | --- | --- |
|  | Rückenschmerzen | |
|  | Herzkreislauferkrankungen (z.B. Bluthochdruck, Herzkranzgefäßverengung) | |
|  | Asthma/chronische Lungenerkrankungen (z.B. Emphysem, COPD) | |
|  | Krebserkrankung | |
|  | Depressionen, Angststörungen oder andere psychische Leiden | |
|  | Diabetes (Zuckerkrankheit) | |
|  | chronische Nierenerkrankung | |
|  | andere:  [Bitte im Feld rechts eintragen.] |  |

1. **Wie oft hatten Sie in den letzten 12 Monaten Kontakt zu folgenden Ärzt/-innen?** (egal, ob persönlich, per Telefon oder per Videosprechstunde)

[Hinweis: Falls Sie sich nicht an die genaue Anzahl erinnern, genügt eine Schätzung.]

**[Bitte setzen Sie in jeder Zeile genau ein Kreuz.]**

|  | **gar nicht** | **1-mal** | **2 bis 3-mal** | **4 bis 5-mal** | **6-mal**  **oder öfter** | **weiß nicht** |
| --- | --- | --- | --- | --- | --- | --- |
| **Hausarzt/-ärztin** |  |  |  |  |  |  |
| **Fachärzt/-innen***  (z.B. Frauenarzt/-ärztin, Orthopäd/-in)  **ohne Zahnarzt/-ärztin* |  |  |  |  |  |  |
| **Psychotherapeut/-in** |  |  |  |  |  |  |

1. **Sie haben nun einige Fragen zur Videosprechstunde beantwortet. Können Sie sich grundsätzlich vorstellen, die Videosprechstunde selbst zu nutzen?**

|  | ja |
| --- | --- |
|  | nein |

**Vielen Dank für Ihre Teilnahme!**
