# Supplementary material for: Inhibiting and Promoting Factors for the Use of Video Consultations Among Individuals Covered by Statutory Health Insurance in German Outpatient Care: Cross-Sectional Study
Source: J Med Internet Res. 2025 Jun 11;27:e66027. doi: 10.2196/66027 (PMC12198700; doi:10.2196/66027)
Supplement: Multimedia Appendix 2 [file jmir_v27i1e66027_app2.docx]

## **Appendix 2: Correlation/association and effect size of inhibiting factors.**

|  | sex* | | age groups** | | community size** | | occupation* | | chronic disease* | |
| --- | --- | --- | --- | --- | --- | --- | --- | --- | --- | --- |
|  | **P** | **effect size** | **p** | **effect size** | **p** | **effect size** | **p** | **effect size** | **p** | **effect size** |
| I have not yet been offered a video consultation. | n.s. |  | <0.001 | 0,073 | n.s. |  | <0.001 | 0.077 | n.s. |  |
| My Internet connection isn't good. | 0.035 | 0.048 | <0.001 | 0.122 | <0.001 | -0.1 | <0.001 | 0.159 | <0.001 | 0.114 |
| I don't have the technical equipment. | n.s. |  | <0.001 | 0.206 | <0.001 | -0.058 | <0.001 | 0.221 | <0.001 | 0.178 |
| I lack experience in the handling technical devices. | n.s. |  | <0.001 | 0.282 | <0.001 | -0.077 | <0.001 | 0.253 | <0.001 | 0.201 |
| I fear that the quality of medical care will suffer. | n.s. |  | <0.001 | 0.093 | n.s. |  | <0.001 | 0.099 | <0.001 | 0.088 |
| I have concerns about data protection. | n.s. |  | <0.001 | 0.128 | n.s. |  | <0.001 | 0.097 | <0.001 | 0.090 |
| I struggle to communicate in German language. *** | n.s. |  | <0.001 | 0.086 | n.s. |  | <0.001 | 0.168 | n.s. |  |
| I find video consultations too exhausting. | n.s. |  | <0.001 | 0.136 | 0.002 | -0.043 | <0.001 | 0.164 | <0.001 | 0.1 |

** chi square test with Cramer’s-V effect size; ** Kendall's-Tau-c; *** only for non-native speakers; The level of statistical significance is set at α=0.05 (p ≤ 0.05).*
