# Supplementary material for: Inhibiting and Promoting Factors for the Use of Video Consultations Among Individuals Covered by Statutory Health Insurance in German Outpatient Care: Cross-Sectional Study
Source: J Med Internet Res. 2025 Jun 11;27:e66027. doi: 10.2196/66027 (PMC12198700; doi:10.2196/66027)
Supplement: Multimedia Appendix 3 [file jmir_v27i1e66027_app3.docx]

## **Appendix 3: Correlation/association and effect size of promoting factors.**

|  | sex* | | age groups** | | community size** | | previous VC experience* | | occupation* | | chronic disease* | |
| --- | --- | --- | --- | --- | --- | --- | --- | --- | --- | --- | --- | --- |
|  | **p** | **effect size** | **p** | **effect size** | **p** | **effect size** | **p** | **effect size** | **p** | **effect size** | **p** | **effect size** |
| Information services | 0.040 | 0.048 | n.s. |  | n.s. |  | n.s. |  | <0.001 | 0.068 | 0.008 | 0.058 |
| Information from my physician about aspects to consider during the video consultation | n.s. |  | 0.019 | -0.031 | n.s. |  | n.s. |  | <0.001 | 0.057 | n.s. |  |
| Easy/intuitive operation of the video application | <0.001 | 0.070 | <0.001 | -0.104 | n.s. |  | <0.001 | 0.070 | <0.001 | 0.127 | 0.005 | 0.059 |
| Video application with many features | n.s. |  | <0.001 | -0.131 | n.s. |  | <0.001 | 0.047 | <0.001 | 0.12 | <0.001 | 0.104 |
| Video application works without interruptions | 0.012 | 0.054 | <0.001 | -0.143 | n.s. |  | <0.001 | 0.077 | <0.001 | 0.156 | <0.001 | 0.073 |
| Can also be used by people with visual, hearing or movement impairments | <0.001 | 0.099 | <0.001 | -0.147 | n.s. |  | <0.001 | 0.068 | <0.001 | 0.121 | <0.001 | 0.072 |
| Information about data protection | <0.001 | 0.069 | n.s. |  | 0.041 | 0.026 | n.s. |  | <0.001 | 0.076 | n.s. |  |
| Conversation support in native language*** | n.s. |  | <0.001 | -0.170 | n.s. |  | n.s. |  | n.s. |  | n.s. |  |

** chi square test with Cramer’s-V effect size; ** Kendall's-Tau-c; *** only for non-native speakers; The level of statistical significance is set at α=0.05 (p ≤ 0.05).*
